# Making Deception Fun: Teaching Autistic Individuals How to Play Friendly Tricks

**DOI:** 10.1007/s40617-024-00935-z

**Published:** 2024-04-10

**Authors:** Megan St. Clair, Kacie Massoudi, Jonathan Tarbox, Adel Najdowski, Lauri Simchoni, Marianne Jackson, Angela Persicke

**Affiliations:** 1 Chicago School of Professional Psychology, Los Angeles, CA USA; 2 California State University, Fresno, CA USA; 3 Center for Autism and Related Disorders, Inc, Woodland Hills, Los Angeles, CA USA; 4 Halo Behavioral Health, Sherman Oaks, CA USA; 5 University of Southern California, Los Angeles, CA USA; 6 Pepperdine University, Malibu, CA USA

**Keywords:** Autism, Deception, Multiple exemplar training, Perspective taking, Theory of mind, Trick

## Abstract

Perspective taking is a critical repertoire for navigating social relationships and consists of a variety of complex verbal skills, including socially adaptive forms of deception. Detecting and being able to use socially adaptive deception likely has many practical uses, including defending oneself against bullying, telling white lies to avoid hurting others’ feelings, keeping secrets and bluffing during games, and playing friendly tricks on others. Previous research has documented that some Autistic^1^ children have challenges identifying deception and playfully deceiving others (Reinecke et al., 1997). The current study employed a multiple baseline across participants design to evaluate the use of multiple exemplar training, rules, modeling, practice, and feedback for teaching four Autistic children and adolescents to use deception to play friendly tricks on others. The procedure was successful for all participants, and generalization was achieved across novel, untrained tricks.

Autism spectrum disorder (ASD) is characterized by childhood social development that differs from the neurotypical population. Successful social interactions in neurotypical culture often depend on taking the perspective of another person. Perspective taking involves verbal responding to the relation between self and others (Hayes et al., 2001), such that an individual can (1) discriminate between their own private events and the potential private events of others; and (2) predict the future behavior of someone else based on this discrimination (LeBlanc et al., 2003).

Accurately identifying and attributing the true and false beliefs of others is a component of perspective-taking skills (LeBlanc et al., 2003). A false belief is when someone thinks something to be true that is, in reality, false (e.g., I might think my doll is in her dollhouse because that is where I always keep her; however, if my brother moved my doll, my belief that it is in the dollhouse is a false belief). Detecting true and false beliefs in others directly relates to understanding and using deception, such that deception involves creating a false belief in another person.

Autistic advocates have identified honesty as a strength of Autistic culture and have explicitly spoken out against Autistic individuals needing to act dishonestly in order to “fit in” in neurotypical social culture (Autistic Science Person, 2021). In addition to valuing honesty as a strength of many Autistic individuals, it may be clinically useful to empower Autistic individuals to use deception when they want to for practical social purposes. For example, it is important to understand when one is being deceived as well as to use deception adaptively, such as when keeping secrets and surprises, bluffing during games, telling white lies, and playing tricks on others. Although some forms of deception may have malicious intent and may be used to take advantage of others, many other forms of deception can be completely harmless and even purposeful, practical, or playful. For example, bluffing in a game is considered a purposeful form of deception because it involves implementing a specific strategy in an effort to win a game. Moreover, white lies may be considered practical because sometimes being truthful can hurt someone’s feelings (Bergstrom et al., 2016). For example, if a child’s friend arrives at school with a new pair of shoes that the child does not like, telling his neurotypical friend that the shoes are ugly may not help maintain their friendship.

Finally, playing friendly tricks on others is a form of deception that can potentially have a positive impact on the development of social relationships with others. Playing tricks on others involves using deceptive statements or contriving false scenarios to make another person believe that something is true when, in fact, it is false for the purposes of being humorous and having fun. This form of deception necessitates the speaker inferring abstract interpretations of the listener’s private events by discriminating between the truth and what the listener is likely thinking (e.g., “Jimmy thinks ink was spilled on the carpet, but it was actually invisible ink”), and in turn, between what one says to that person and the actual truth (e.g., “Look, there’s a spider,” but it is actually a fake spider).

Research conducted with neurotypical children has found that as young as 2 and 3 years old they begin practicing deception and by 4 years old they have a concrete understanding of false beliefs and begin to use deceptive ploys by playing tricks on others (Sodian et al., 1992). On the other hand, research has demonstrated that some Autistic children have challenges in perspective taking, understanding false beliefs, identifying deception, and using deception to play tricks on others in a friendly social context (Baron-Cohen et al., 1985; Perner et al., 1989; Talwar et al., 2012).

A majority of the research on false beliefs stems from the Theory of Mind (ToM) literature and is associated with assessments designed to determine whether Autistic children identify the false beliefs of others or engage in deception. The classic test of false-belief identification is the Sally-Anne Task or unexpected transfer task (Baron-Cohen et al., 1985). In this task, there are two characters, Sally and Anne. Sally places a marble into a basket and then leaves the room. While Sally is gone, Anne moves the marble to a box. Upon Sally’s return, the participant is asked to identify where Sally will look for her marble. If the participant says Sally will look in the box, the response is scored as an error in identifying Sally’s false belief that the ball is where she put it. Research has found that Autistic participants often answered the question of where Sally would look for her marble in accordance with their own perspective instead of Sally’s perspective (Baron-Cohen et al., 1985). The Sally-Anne task is relevant to deception in the form of playing tricks because, essentially, Anne was tricking Sally by misplacing her ball. If Autistic individuals do not identify when a trick is being played within a social context, it seems likely that they may also have difficulty with playing a trick on someone else, although this is an empirical question that has not yet been addressed by research.

Another false-belief task used in traditional psychology involves a deceptive container task, also known as the Smarties test (Hogrefe et al., 1986; Perner et al., 1987). In this task, an item other than what is expected is placed inside a commercial package (e.g., a pencil is placed inside a Smarties candy box) after a third person leaves the room. Upon return, the participant is asked to identify what the third person will think is in the package. This task is relevant to deception in the form of playing tricks because a participant must identify that others will develop a false belief under circumstances in which a particular situation has changed without their knowledge. In designing and developing tricks to play on others, the participant must learn to systematically and discretely arrange those conditions to create false beliefs in others, much like the experimenter does when placing an unconventional item in the commercial package when conducting the Smarties test.

Behavioral intervention has been successful in teaching Autistic individuals to identify the false beliefs of others using the Sally-Anne task and deceptive container task arrangements. For example, Charlop-Christy and Daneshvar (2003) and LeBlanc et al. (2003) used video modeling to teach participants to pass false belief tests, whereas Dhadwal et al. (2021) taught this skill during in-vivo contrived situations presented in the natural environment. A limitation of these studies is that they did not measure whether participants applied what they learned to social interactions. In other words, it is unknown if participants went on to use the skill to create false beliefs in others or to identify when others were deceiving them.

Given how prominent perspective taking is in everyday interactions and considering the specific social and communicative functions underlying an understanding of false beliefs, finding effective procedures for teaching Autistic individuals to understand deception seems warranted. Although substantial research has documented that some Autistic individuals have challenges with understanding and using deception, only a few studies have attempted to teach it directly. For example, Bergstrom et al. (2016) used behavioral skills training (BST) to teach Autistic children to tell polite “white lies” when given an unwanted gift or when another person’s appearance changed in an undesirable way.

In addition, two studies have focused on teaching children to play tricks. For example, Russell et al. (1991) used a “windows task” in an attempt to teach Autistic children to strategically trick an experimenter into thinking chocolates were in boxes that were actually empty. First, each participant was instructed to direct the experimenter where to look for chocolate that was hidden in one of two boxes placed between them. During this phase of training, the participant learned what would happen based on whether they accurately or inaccurately directed the experimenter. If the experimenter accurately located the chocolate based on the participant’s directions, then the experimenter got to keep it; however, if the experimenter was led by the participant to choose the empty box, then the participant kept the chocolate for themself. In the second phase, identical boxes were used, except that they were constructed with windows that faced the participant in order for the participant to clearly identify which box contained the chocolate and which box did not. With little additional prompting, neurotypical participants and participants with Down’s syndrome quickly learned that it was in their best interest to deceive the experimenter by tricking the experimenter into looking in an empty box for the chocolate; however, the Autistic participants frequently continued to point to the box containing chocolate and did not acquire the skill of tricking the experimenter.

In another study, Reinecke et al. (1997) used a multiple baseline across participants design to assess the effects of a procedure that attempted to teach Autistic adolescents to use deception skills within a game-play context. The procedure involved a training package of rules, modeling, role-play, and immediate feedback. Using this training package, participants were taught to hide a penny (in one of their hands) from an observer who then guessed the hand in which it was hidden. The dependent variable used to measure the efficacy of the training package was the deception skills employed by each participant. These deception skills included object occlusion, hidden transfer, empty fist closed, hiding fist closed, and not indicating tactics to “trick” the experimenter into choosing the wrong hand. The procedure was effective for the first participant but the other two participants learned the response during baseline, which made the internal validity of the study questionable.

Outside of these studies, teaching adaptive deception skills is largely unstudied in the behavioral research literature. Therefore, the purpose of the current study was to extend behavioral research in this area by evaluating a training package that included multiple exemplar training (MET), rules, modeling, practice, and feedback (e.g., praise and error correction), for teaching four Autistic children and adolescents to use deception to play friendly tricks on others. The use of treatment packages that include MET and rules has been effective for teaching other perspective-taking skills such as detecting sarcasm (Persicke et al., 2013), social conflicts (Suarez et al., 2021), responding to disguised mands (Najdowski et al., 2017), and responding to the preferences of others during play (Najdowski et al., 2018).

## Method

### Participants, Settings, and Materials

Participants included four Autistic children and adolescents. Charlie was a 16-year-old caucasian male who received approximately 10 hr per week of one-to-one behavioral intervention and had been receiving in-home applied behavior analytic intervention for approximately 6 years. English was his primary language, and he lived in an upper-middle-class neighborhood. Doug was a 9-year-old caucasian male who received approximately 6 hr per week of small group social skills training and had been receiving behavior analytic services for approximately 6 years. Of those years, he initially received 3 years of early intervention and subsequently 3 years of social skills training with one-to-one support. English was his primary language, and he lived in a middle-class neighborhood. James was an 8-year-old caucasian male who received approximately 20 hr per week of one-to-one behavioral intervention and had been receiving in-home applied behavior analytic intervention for approximately 1.5 years. English was his primary language, and he lived in a lower-middle-class neighborhood. Chris was a 9-year-old Latinx male who received approximately 2 hr per week of small group social skills training and had been receiving applied behavior analytic intervention for approximately 7 years. Of the 7 years, approximately 4 were in early intervention, and the remaining 3 were in social skills training. English was his primary language, and he lived in a middle-class neighborhood.

All participants had well-developed language skills, which included echoics, mands, tacts, and intraverbals. They spoke in complete sentences and engaged in back-and-forth conversation. They followed rules and exhibited a generalized imitation repertoire. In addition, all participants had mastered identification of what others are sensing (i.e., accurate identification of what others could see, hear, smell, taste, and feel when asked, “What do / does [*person / pronoun*] [see/hear/smell/taste/feel]?”; e.g., “What does mom smell?” “Popcorn!”; Welsh et al., 2019) and cause and effect (i.e., identification of potential causes when asked about visually apparent effects on stimuli in the environment, “Why [*explanation of the effect*]?” “Because [*explanation of the cause*]”; e.g., “Why is the glass frame broken?” “Because it was knocked off the shelf”; as well as identified and predicted potential effects of what might have happened when given an explanation of the cause,“What happened / will happen when / if [*explanation of cause*]?; e.g., “What will happen if I stick this pin in the balloon?” “It will pop!”). Moreover, predicting emotional causes (i.e., “What would make [*person / pronoun*] feel [*emotion*] in [*situation*]?”; e.g., “What would make your older brother so angry that playing the trick would no longer be fun?”) and effects (e.g., “How would [*person / pronoun*] feel if [*situation*]?”; e.g., “How would your grandmother feel if you played a trick on her that included you or someone else being very hurt?”) were prerequisite skill repertoires for discriminating “mean” versus “nice” tricks.

To be included in the current study, participants’ parents, clinical supervisors, and clinical team needed to regard playing tricks as clinically relevant and as an important and necessary target skill for each participant’s overall treatment and development. Playing friendly tricks was considered a clinically relevant skill to target when potential participants had a history of attempting to perform such tricks on others, but failing to do so successfully because they were missing a required component of trick-playing behavior. For example, a participant might giggle and smile uncontrollably while playing a trick or might not have been aware that most of their body was visible while trying to hide from someone.

All participants included were also required to have an active motivating operation for successfully playing tricks on others, as evidenced by either their direct report or observation of repeated, but ineffective, demonstrated efforts. In this way, each participant presented with a desire to play a trick on others, but did not effectively do so. That is, in addition to parents and clinicians stating that playing tricks was an important target of intervention to them, the participants themselves indicated that they wanted to play tricks on others. None of the participants had ever received previous direct training on how to use deception to play tricks on others, and throughout the course of the study, playing tricks was not targeted outside of the study by anyone.

All sessions were conducted within the context of normally scheduled, ongoing behavioral intervention or social skills training sessions, either in the participant’s home or in a clinic. Only one session was conducted daily, with one to four weekly, ranging in duration from 1 to 2 hr. Each session, three to seven trick trials were conducted and each trial was approximately 10 min in duration.

### Response Measurement and Interobserver Agreement

Playing a trick was defined as any instance in which the participant independently completed each of the following components of trick-playing behavior: (1) *stating*; (2) *executing*; (3) *inhibiting*; and (4) *ending* the trick.

In order for *stating* responses to be scored as correct, the participant had to describe the trick to be played and initiate an explanation of the deception component (within 5 s of being asked) by discussing how they were going to do something or what they were going to tell someone that was not actually true, but would make the person think was true (e.g., “Let’s play a trick on Mom where I hide her cell phone and then ask her to use it to play a game. Mom will think it is where she last left it and will look for it there, but really it will be where we hid it!”). During phase 1 of training, if the participant engaged in no response, did not describe a trick to be played, and/or did not explain the deception component appropriately, the stating response was scored as incorrect. In addition to these response requirements, during phase 2 of training, if the participant did not describe a *novel* trick to be played and/or described a previously generated trick to be played with the same person it was already executed on, the stating response was scored as incorrect.

*Executing* responses were considered independent from stating. Within 1 min of stating the trick, participants were expected to begin executing that trick for a duration of no longer than 10 min. In particular, if the participant successfully carried out the trick they described (e.g., placing oneself in a closet in preparation for “popping out” at a family member passing by), within 1 min of stating it and for no longer than 10 min, and the individual the trick was being played on reported truly being tricked (e.g., because they had no idea the participant was in the closet and were completely startled), it was scored as a correct execution response.

In order for *inhibiting* responses to be scored as correct, the participant had to suppress any vocalizations, intonation, facial expressions, gestures, and/or body language that would “give away” the trick to the person it was being played on until that person reacted to the trick. In other words, each participant was required to engage in inhibition for the duration of the trick, and the criterion for a correct response required that the other person was, in fact, actually tricked. If the participant laughed; smiled; engaged in nervous or excited fidgeting; discussed the trick too much, too loudly, in too close of proximity to the person to be tricked; and/or gave away the trick in any other individualized manner evident to and reported by the individual being tricked, inhibition was scored as incorrect. Inhibiting responses were considered independent from executing, in that execution instructions involved telling the participant what *to* do, whereas inhibition instructions involved telling the participant what *not* to do.

After each trick, the person who was being tricked was interviewed to determine whether the trick was implemented effectively with successful inhibition of any responses that might alert them to the fact that they were being tricked. The self-report of individuals being tricked was required, because participants would, at times, engage in individualized, discrete behavior that gave away their trick to those who knew them really well, but would have been too subtle or personalized for the experimenter to readily detect. Therefore, such individualized feedback to the participant was valuable in training participants in their own independent execution and inhibition repertoires. As such, all individuals tricked in this study were instructed to respond naturally to participants respective to whether they were really being tricked (e.g., laughter, sigh of relief) or not (e.g., confusion, stating they know it is a trick). Subsequent to the participants' trick attempt, these persons were formally asked, “Were you *really* tricked, or were you on to [*participant*]?” If the person stated that they knew they were being tricked, they were asked to describe what the participant did that gave the trick away. This cued the experimenter to any highly discrete, personal participant responses that represented an execution error from the direct perspective of the individual being tricked and their relation to the participant, as well as assisted the experimenter in customizing the specific corrective feedback to be provided to the participant. For example, if a participant was playing a “made you look” trick on their nanny using a snake prop, but the nanny indicated that the participant’s body language and facial expressions were calm and casual with a flat intonation in their speech, corrective feedback would entail evoking contextually congruent nervous body language, fearful facial expressions, and distressed intonation to help “convince” the other person that they were being truthful, when in fact they were playing a trick.

Finally, an *ending* response was considered correct if it met two criteria. First, a response was scored as correct if the participant made a common ending statement (e.g., “Tricked you!” or “Gotcha!”) to the person within 5 s of seeing them react to the trick. Second, the participant had to explain the deception involved to the experimenter within 5 s of being asked by the experimenter (e.g, “[*Participant*] tell me about the trick we just played.” “I made my sister think she left her homework at school, but really I just took it from her backpack and hid it!”). If the participant did not make an ending statement that matched the purpose of the trick and/or did not accurately explain the deception involved within the trick, the ending response was scored as incorrect.

Data were collected on each of the four trick-playing behaviors within each trial. The percentage correct was calculated for each trial and then averaged across trials to calculate a total percentage correct for each session. Accuracy data on trick trials were summarized and graphed separately as percentage correct.

A second independent observer simultaneously collected data on 47%, 50%, 38%, and 43.75% of all sessions for Charlie, Doug, James, and Chris, respectively. Interobserver agreement scores were determined for each session by calculating the sum of agreements on the occurrence or nonoccurrence of correct trick-playing behaviors and dividing the number of agreements by the sum of agreements and disagreements and multiplying by 100%. Agreement averaged 99%, 100%, 98%, and 95.90% for Charlie, Doug, James, and Chris, respectively.

### Experimental Design and Procedure

A nonconcurrent multiple baseline across participants design was used to evaluate the efficacy of the training package. Sessions were designed to be indistinguishable from participants’ regularly scheduled in-home or clinic-based intervention sessions. Trick trials were conducted during play breaks from other intervention programs. Breaks were provided between each trick trial and were long enough to conduct one or two other skill acquisition programs within the participants' behavioral intervention programs.

### Baseline

During each baseline session, the experimenter instructed the participant to engage in playing a trick on someone (i.e., “Let’s play a trick on someone. Who should it be and what should we do?”). After the delivery of the trick instruction, each participant had 1 min to initiate a trick and 10 min to complete it. If 1 min elapsed and the participant had not initiated a trick by beginning to state the trick or by engaging in behaviors to play a trick, all four components of trick-playing behavior were scored as incorrect, and the participant was redirected to another activity before moving on to the next trick trial. If the participant initiated a trick, but 10 min elapsed before the participant completed the trick, the individual components of trick-playing behavior that were completed were scored as either correct or incorrect and any uncompleted components were scored as incorrect. No prompting was used in baseline and no programmed consequences were provided for any participant's responses to the instruction to engage in playing a trick. Throughout baseline, sessions were no longer than 1 hr in duration, wherein a total of three trick trials were conducted.

### Training

Throughout training, three to seven trick trials were conducted per session, and all sessions ranged in duration from 1 to 2 hr. During training trials, experimenters honored the participants’ choice to assent to the procedures or not. Escape extinction was not implemented and participants were not required to comply with training. In particular, if a participant indicated that they did not want to play a particular trick on someone, the experimenter asked the participant if they wanted to think of a different trick or if they wanted to think of a different person to play a trick on. If a participant vocally indicated that they did not want to play any tricks on anyone or nonvocally withdrew assent by engaging in any behavioral escalation, the training trial would have been terminated; however, this never occurred.

#### Phase 1: Rules, Discrimination Training, Modeling, and Contrived Practice

Phase 1 of training included all of the following components presented sequentially in order for participants to learn the basics of successfully completing a trick: rules with example and nonexample, modeling, and contrived practice. This phase was carried out until an effect was apparent through visual inspection of data.

##### **Rules and Discrimination Training**

At the beginning of session 1, the participant was provided with the rule, “Sometimes people play tricks on one another for the purposes of teasing the other person and having fun. A trick is when someone says something or does something that is not really true, but tries to make another person think it is true, even though it isn’t.” Then, the participant was given a randomly ordered example and nonexample to assist with discriminating a trick from a nontrick (e.g., “For example, I would be tricking you if I told you that you had a big, ugly stain on your shirt, because you don’t, but if you *did* have a stain on your shirt it wouldn’t be a trick because it would be true.”). Immediately upon the provision of the example and nonexample, participants were asked follow-up questions to ensure attending and rule comprehension (e.g., “So, why would telling you that you have a big, ugly stain on your shirt b*e a trick*?” “Because I don’t have one / Because it isn’t true!” and “Why would telling you that you have a big, ugly stain on your shirt *not be a trick*, if your shirt really was actually stained?” “Because I do have one / Because it is true!”). Correct follow-up answers resulted in praise. Incorrect follow-up answers resulted in error correction in the form of the provision of the correct interpretation (e.g., “Telling you that you have a big, ugly stain on your shirt *would be* a trick because you do not really have one!” and “Telling you that you have a big, ugly stain on your shirt if your shirt really was stained *would not be* a trick because then you would actually have a stain and it would be true!”). Additional novel and arbitrary examples and nonexamples continued to be discussed until the participant correctly discriminated between a trick versus a nontrick within one presented trick scenario.

After providing one correct, independent discrimination of a trick versus a nontrick, session 1 continued by instructing participants to differentiate between mean versus nice tricks using the rule, “Remember, playing tricks is for fun and to make people laugh! Tricks should not be played if they hurt someone’s feelings or if they cause something to break that cannot be fixed.” Next, the participant was required to identify a potentially mean trick (e.g., “Can you think of any *mean* tricks?”), as well as explain why it is considered mean (e.g., “Why would that trick be *mean*?”). When the participant accurately identified one trick that would be mean and explained why, they were told that they were correct and praised. If the participant did not accurately identify and explain a mean trick, the experimenter modeled an example (e.g., “For example, a trick would be mean if we really stained someone’s shirt, because we might not be able to fix it and that person’s shirt would be ruined!”). Then, the experimenter asked the participant to present their own example. This continued in a back-and-forth manner until the participant ended on a correctly generated example of a mean trick and explained why it was mean. The same procedure was used for identifying a nice trick. Once participants could identify and explain at least one novel mean and nice trick example, this phase of discrimination training with social rules was considered complete.

##### **Modeling**

Next, a nice trick was modeled by the experimenter in order to demonstrate what an appropriate trick would look like when conducted with another person (see Table 1 for categories of tricks and examples of tricks in each category). At least one of each type of trick was modeled for each participant; however, there was no direct instruction given to participants about the different categories of tricks. First, *stating* was modeled (e.g., “Playing a trick on someone looks something like this: “[*Participant*] I’m going to tell [*person*] we broke a vase, even though we did not break a vase, because I know she would not like for her vase to be broken and she might freak out! [*Person*] will think we broke her vase, but really her vase will be fine and she will be so relieved when we show her!”) using inhibition (e.g., whispering or relocating if the person with whom the modeled trick was to be played was near). Second, *executing* was modeled according to the plan by addressing the person with whom the trick was to be played (e.g., “[*Person*] we broke your vase!”) in a panicked tone of voice, with tense body language, and a concerned facial expression. Third, *inhibiting* was modeled by clearly withholding any laughing, smiling, or talking about the trick in a way that could give away the trick. Fourth, *ending* the trick was modeled by letting the third person know that they were being tricked (e.g., “Gotcha!”) and subsequently explaining the deception just modeled (e.g., “We made [*person*] think we broke her vase, but really, her vase was not broken!”).

**Table 1:** Examples of Tricks in Each Category

Category	Example
“Made you look!” / Fake Out	Saying something is there or something has happened that is not really there or has not really happened (e.g., “Look there’s a spider crawling on your pants!”; giving a terrified facial expression while pointing and saying, “Look! Do you see it? Right there! Look!”; “How did your vase break?”; planting a fake snake in a sibling’s shoe).
“Surprise!” Pop Out	Hiding somewhere or having something hidden somewhere that pops out in surprise with a “Boo,” other jarring sound, or unanticipated movement (e.g., hiding under the bed and grabbing someone’s feet; setting up a “booby trap” of stuffed animals ready to fall from a net when the door is opened; offering a “candy container” prop that really has a pop-out clown when opened).
Friendly Fib	Telling someone something that is not really true (e.g., “Quick! The toilet is overflowing!”; “Dad, the basketball cracked the windshield of the car!”; “My school principal gave me detention”).
Secret Sabotage	Altering someone’s belongings in some way that deviates from their expectations (e.g. putting a silly selfie on Mom’s phone when she’s not looking; switching the socks to the underwear drawer and vice versa; removing batteries from a television remote control).
Hidden Object	Hiding something from someone who is going to need to use it or is about to look for it in a place where they would normally find it (e.g., moving someone’s only pencil off their desk when they leave their desk to go to the restroom; hiding keys from their typical location when the driver is ready to leave; removing the salt shaker from the table when needed for an upcoming meal).

##### **Contrived Practice**

After one trick was modeled, contrived practice was conducted to afford participants opportunities to carry out a trick. During contrived practice, participants were expected to independently follow through with each of the four trick-playing behaviors using either previously learned tricks (e.g., those modeled) on the same person for continued practice, a previously learned trick with a novel person, or novel tricks that they thought of but had never played before. There were no requirements put in place regarding which categories (displayed in Table 1) of tricks the participants should carry out, as we wanted them to have the autonomy to choose the tricks they wanted to perform, based on their personal preference.

The participant was instructed to play a trick on someone who was physically present in the home or clinic but was not the experimenter (e.g., “Let’s play a trick on someone, who should it be and what should we do?”). After the delivery of each trick instruction, each participant had approximately 1 min to initiate the trick they stated and 10 min to complete the trick, just as in baseline. When participants responded correctly on all four components of a trick, they received descriptive verbal praise (e.g., “You did it! You played a very funny trick and [*person*] really believed [*false statement for trick*] even though it wasn’t true! Great job!”) and brief access to a preselected preferred item (5 min or less) or activity (10 min or less). Preferred items or activities were determined via an informal preference assessment, wherein participants were provided with choices of items or activities eligible for earning, from which they made their preferred selection.

For incorrect responses, error correction procedures were implemented. Error correction involved prompting the participant to identify the steps of the trick performed incorrectly and how to respond appropriately in later trials in a least-to-most intrusive presentation (i.e., experiential–leading question–role play–partial vocal directive–full vocal directive). In particular, *experiential* prompts permitted the participant to make mistakes during execution, while the experimenter prompted the person the trick was being played on to respond appropriately (e.g., by showing that they were not tricked). This afforded the opportunity for the participant to undergo the natural consequences of their misperformed trick. *Leading question* prompts included asking the participant questions such as, “What did you forget to do?” or “Why do you think [*person]* didn’t fall for the trick?” *Role-play* prompts involved practicing each trick-playing behavior aloud to identify if the participant could detect errors when reenacted. *Partial vocal directive* prompts included giving the participant a hint that they were missing a trick-playing step or implementing it inaccurately, for example, “You forgot something!” or “There is something you need to fix to make the trick happen.” Finally, *full vocal directive* prompts involved directly explaining to the participant which trick-playing step(s) was performed incorrectly and giving an explanation for why.

#### Phase 2: Rules, Contrived Practice, and MET

During phase 2 of training, discrimination training and modelin*g* were discontinued and the remaining sessions of training included the following components presented simultaneously: rules, contrived practice, and MET. MET (Erhard et al., 2021) was conducted to establish the generalized behavior of creating and playing tricks across multiple exemplars. Within this teaching phase, participants were expected to differentially and independently follow through with each of the four trick-playing behaviors using only novel tricks the participant designed and had never played before or a previously learned trick with a novel person they had never played it on before. In this way, MET was implemented to promote the acquisition of a trick-playing repertoire, rather than rote, rigid, or repetitive acquisition of a particular trick or trick type.

The following instruction was provided to participants: “Let’s play a trick on someone that we have never played on them before, who should it be on and what should we do?” Unlike in phase 1, to be scored as correct, participants could no longer repeat any previously learned tricks with the same person during phase 2. In this manner, this phase took into consideration the trick-playing history of the participant with others and how this could affect successful deception. For example, if a participant repeatedly went up to the same person, pointed to any piece of their clothing (e.g., shirt the first time and pants the next), and said, “Oh no! What happened?” (i.e., suggesting a stain), eventually the person may respond, “Not this *again*!” in recognition that they are being tricked in the same way they already have been before. The same reinforcement and error correction procedures described in the contrived practice section of phase 1 of training were employed in phase 2. During phase 2, data were collected until participants’ responding was considered stable at or near 90%–100% correct upon visual inspection.

### Social Validity

At the conclusion of training, parents were asked to complete a seven-item survey to provide written feedback regarding the overall acceptability of the clinical procedures that were used to teach their child or adolescent to play friendly tricks on others and the total satisfaction with treatment outcomes. This measure was designed to assess potential positive and adverse effects the study may have had on participants. For six of the seven questions, parents were asked to provide a rating on the scale of 1 (strongly disagree), 2 (disagree), 3 (neutral), 4 (agree), or 5 (strongly agree). Question 1 stated, “I am happy with the procedures that my child’s clinical treatment team used to teach them how to play friendly tricks on others.” Question 2 stated, “My child enjoyed learning how to play friendly tricks on others.” Question 3 stated, “My child benefited from learning how to play friendly tricks on others.” Question 4 stated, “I think that the ability to play friendly tricks on others was important for my child to learn.” Question 5 stated, “I would recommend the playing tricks training program implemented by my child’s treatment team to other parents who have a child with autism.” Question 6 stated, “The playing tricks training program did not have any negative side effects.” The final question was open-ended and stated, “Is there any additional feedback you would like to add regarding your child’s participation in the playing tricks training program?”

## Results

**Fig. 1:**
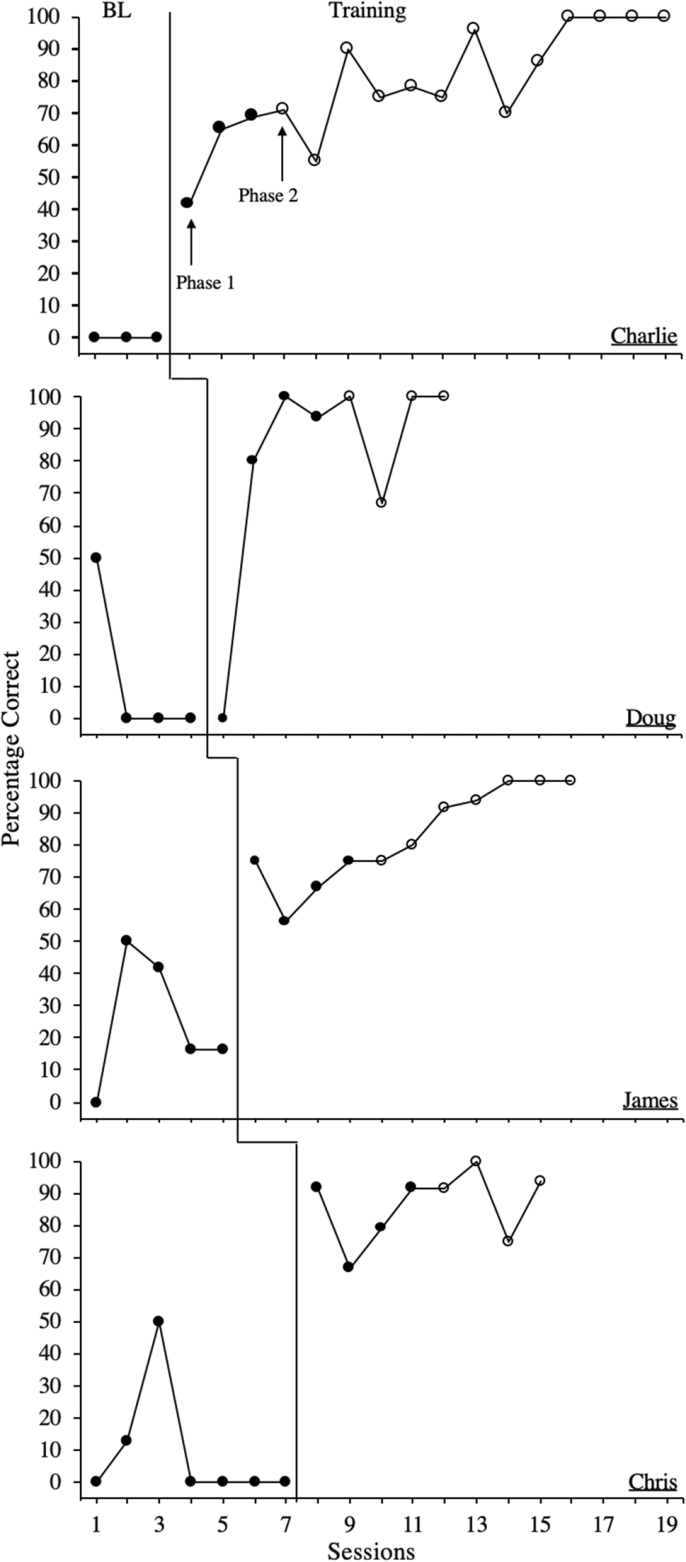
Percentage of Correct Trick-Playing Behavior. *Note.* Overall percentage of trick-playing behaviors implemented correctly across baseline and training. During the training phase, the closed circles denote phase 1 of contrived practice and open circles denote phase 2 of contrived practice

Figure 1 depicts the overall percentage of trick-playing behaviors performed correctly across baseline and training. During baseline, Charlie’s (first panel) accuracy was at zero, as he did not demonstrate any of the four trick-playing behaviors required to effectively play a trick on others. However, upon implementing phase 1 of training, there was an immediate increase in level and trend. Further, upon advancing training to phase 2, 100% accuracy was achieved during the thirteenth training session and maintained for the remaining three sessions.

During baseline, Doug (second panel) responded with 50% accuracy during the first session, which dropped to zero for the remaining baseline sessions. Upon implementing phase 1 of training, Doug scored zero for the first session, followed by an immediate increase in level and trend. During phase 2 of training, Doug responded with 100% accuracy during the first session but decreased to 67% before immediately returning to 100% accuracy across the two remaining sessions.

During baseline, James (third panel) responded with 0%–50% accuracy, demonstrating some of the component trick-playing behaviors. Immediately upon implementing phase 1 of training, there was an increase in level. Then, upon advancing phase 2 of training, accuracy in responding continued to improve with a gradual increasing trend. James demonstrated 100% accuracy during the ninth training session, which maintained for the remaining two sessions.

During baseline, Chris (fourth panel) responded with 0%–50% accuracy, demonstrating some of the component trick-playing behaviors. Immediately upon implementing phase 1 of training, there was an increase in level. Then, upon advancing to phase 2 of training, Chris continued to respond with high levels of accuracy (X̅ = 90.10%, range: 75%–100%).

Three of the four participant’s parents completed the social validity questionnaire. The fourth participant’s parent did not return the completed questionnaire. Two of the parents gave the highest acceptability rating of 5 (strongly agree) for all of the Likert-scale questions. Both of these parents also indicated on the open-ended question that the clinical procedures had additional gains outside of those that were programmed. In particular, Charlie’s parent reported, “We’ve seen a lot of generalization and I think this has really helped [Charlie] to understand the concept of knowing.” James’s parent reported, “It was a really fun, engaging way for [James] to learn a number of social skills.” The third caregiver who filled out the social validity questionnaire gave a rating of 4 (agree) for all but one item (“My child enjoyed learning how to play friendly tricks on others”) which received a rating of 3 (neutral).

## Discussion

A training package consisting of MET, rules, modeling, practice, and feedback (e.g., praise and error correction) increased correct trick-playing behavior for all four participants. Generalization was observed across novel, untrained tricks that were independently developed and implemented by participants, therefore suggesting that trick-creation and trick-playing can potentially be strengthened as a larger generalized operant (Dixon et al., 2021). Social validity measures found that parents believed the goals, procedures, and outcomes to be acceptable. Furthermore, inclusion of the criterion of persons being tricked having to identify if the participant gave away the trick represents a potential strength of the study because unique and subtle participant responses that may expose their trick to those who know their personalized facial expressions, body language, and intonation well, was strategically taken into account.

The findings of this study contribute to the existing behavior analytic research literature on teaching complex social skills and nonliteral language (e.g., Persicke et al., 2013; Bergstrom et al., 2016) by offering a systematic evaluation of a training package for teaching Autistic individuals to use deception to play friendly tricks on others. Therefore, it represents a small step in attempting to fill a gap in the literature on teaching complex social repertoires to individuals with developmental disabilities. More important, trick-playing was found to be an efficacious strategy for teaching Autistic individuals to use deception in a friendly and playful manner, rather than maliciously, and teaching procedures for this complex social skill repertoire within a trick-playing context made acquiring the skill fun.

One potential strength of the procedures evaluated in this study is that they focused on creating joyful and fun interactions between Autistic participants and others, rather than focusing on establishing particular topographies of social behavior that are deemed “appropriate” by the neurotypical majority. If playing friendly tricks on each other is experienced as mutually positively reinforcing between Autistic individuals and their neurotypical peers and family members, then it may help create a context for social interactions that are based around shared positive reinforcement, rather than social conformity.

In addition, the procedures studied here explicitly called for the trick player to identify how their behavior may cause emotional effects on others (e.g., “tricks have to be fun, they can’t hurt the other person’s feelings”), so it seems possible that the procedures studied here could help build skills in other forms of socially adaptive deception and perspective taking, for example, in preparing a surprise present or party for someone. Of course, no data on generalization to other forms of perspective taking were collected, so these possibilities remain purely speculative. Future research could consider attempting to use teaching trick-playing as a springboard for making perspective-taking training more positively reinforcing and therefore helping expand perspective-taking training to broader socially adaptive repertoires.

Very little previous research has attempted to conceptually analyze deception from a behavior analytic perspective. A pair of recent studies on maladaptive lying in typically developing children conceptualized that the consequence of lying is likely an important controlling variable. In particular, Sauter et al., (2020) and Stocco et al. (2021) conceptualized that lying about misbehavior is more likely when the consequence is neutral or there is reinforcement available, whereas lying is less likely when an aversive consequence is probable, and their initial data support this interpretation. This conceptualization would seem to suggest that socially adaptive deception, including playing tricks would be sensitive to the consequences that such behavior would bring about. The behavior of creating and executing novel tricks, which by definition entails not having contacted the consequences in the past, appears highly complex and seems to involve talking about a verbally constructed future that has not happened yet, and that one intends to create with one’s own future behavior. Complex verbal behavior such as this likely involves deriving rules about potential future behavior and its consequences (Skinner, 1969). Tarbox et al. (2020) used relational frame theory (RFT) to analyze the complex verbal behavior likely involved in deriving cause-and-effect relations between potential behavior and potential consequences in deriving rules about potential future behavior. At a minimum, such verbal behavior likely involves relations of coordination between the words for actions and the actual actions, the words for consequences and actual consequences, and causal relations between the words for actions and the words for consequences. In the case of planning tricks, such rule-deriving might follow a format such as “IF deception behavior THEN person will not know. . . .” For example, participants in the study derived rules such as “IF mommy didn’t see me go into the closet, THEN she doesn’t know I’m hiding in here and she’ll be surprised when I pop out.” According to an RFT analysis, this complex verbal relational repertoire should be teachable through MET (e.g., practicing planning many different tricks) and should be able to be brought under relevant antecedent contextual control (e.g., “Let’s play a trick, what should we do?”).

It is worth noting that all participants in this study had well-developed language skills, which included echoics, mands, tacts, and intraverbals, they spoke in complete sentences, engaged in reciprocal conversation, followed rules, could identify what others are sensing, could identify emotional cause-and-effect relations between behaviors and emotions (e.g., “Why is Sally sad—because Jimmy was mean to her”), and predicting emotional causes (i.e., “What would make Sally happy in this situation?”). It is possible that some or all of these repertoires were necessary prerequisite skills for the current training to be effective. Future research could consider identifying and assessing the necessary and sufficient prerequisite skills for the current training package to be effective.

Although these results are promising, this study was not without limitations and there remain questions to be addressed in future research. First, teaching procedures included only a brief discrimination training component requiring participants to independently identify a mean versus nice trick when provided with a simple social rule and modeled examples as supplementary prompts. This teaching phase encouraged the initial development of some basic verbal discrimination skills involved in trick-playing behavior; however, future research could include other relevant and nuanced social rules. For example, what makes a trick mean, nice, or appropriate, will depend on the person, their preferences, sensitivities, context, environment, and so forth. Successful trick playing therefore likely involves some amount of perspective taking with respect to the person being tricked. Previous research has illustrated that participants with ASD could be taught to identify others’ preferences (Najdowski et al., 2018). Future research could incorporate a similar methodology when teaching identification of others’ preferences and sensitivities as a component of identifying appropriate tricks to play. For example, if Mom’s favorite vase was handed down to her from her grandmother, playing a trick that the vase was broken (when it was not), may not be a good idea, as it could be considered mean in this case.

Second, there was no formal data collection on the discrimination training component within phase 1. In particular, data were not collected on participants' acquisition of their independent discrimination with rule comprehension of both a trick versus a nontrick, as well as discrimination with explanation of a mean versus nice trick. Moreover, only one independent correct discrimination with comprehension and explanation were required for participants to move forward to the next component of intervention. Although results did show that this was sufficient for participants within this study, they may vary across learners and their individual skill acquisition rates within building complex social skills that require perspective taking. Future research could more systematically evaluate discrimination training with rule comprehension and explanation through data collection and analysis to assess effectiveness of teaching procedures and mastery criterion across learners. Related to this, some may be concerned that participants could fail to effectively learn the discrimination between tricks that are fun versus those that are mean or harmful to others, that is, some participants could play tricks that “go too far.” In order to assess this possibility, the social validity assessment explicitly asked caregivers whether any negative side effects were observed, to which all caregivers answered in the negative.

A third limitation of this study is that procedural integrity data were not conducted. Further research should collect procedural integrity data to systematically analyze the extent to which the intervention was being performed correctly. This type of data collection would have better secured treatment fidelity, protected against procedural drift, and afforded opportunities for it to be promptly identified and corrected should such drift have occurred. However, trained board certified behavior analysts (BCBAs) of 7 to 9 years with extensive previous experience in teaching complex social skills to Autistic individuals, both within applied research and experimental sessions, as well as within direct service delivery and treatment as usual, conducted all of the sessions across participants for the current study.

Fourth, maintenance was not evaluated. Future research should evaluate if accuracy in using deception to play tricks on others remains intact during follow-up probes. Moreover, reinforcement schedule thinning could be systematically programmed to gradually remove contrived reinforcers, while also carefully evaluating maintenance of treatment gains under the natural social reinforcement of playing tricks with others.

Fifth, recalling events was not assessed as a necessary prerequisite, but may have led to incidental incorrect responding during phase 2 of training (i.e., participants may have suggested playing the same trick on the same person because they had forgotten that they had already done it once before). Future research should more closely examine the prerequisites necessary to learn this skill and how acquisition is affected by the presence or absence of particular skills. Furthermore, we did not assess whether participants exhibited the skill of identifying information others know or do not know and why they know or do not know the information prior to conducting this study. This “knowing” skill seems relevant to teaching deception because presumably one identifies that someone will not know information and will therefore be tricked. Albeit anecdotal, at the conclusion of the present study, caregivers and clinical treatment team members reported the emergence of this skill during trick-playing teaching procedures without explicit training. Although teaching the identification of what others know has been targeted directly in previous research (St. Clair et al., 2022), future research could more closely examine the possibility of incidental acquisition of identifying what others know within a trick-playing context.

Sixth, participants were taught to respond to the questions, “Let’s play a trick on someone, who should it be on and what should we do?” (training phase 1) or “Let’s play a trick on someone that we have never played on them before, who should it be on and what should we do?” (training phase 2) across sessions. Therefore, it is possible that trick-playing behaviors only came under the stimulus control of these questions and the presence of an experimenter and might not be spontaneously emitted outside of the research context. However, it was anecdotally noted by parents and behavior technicians on the participants’ treatment teams that participants began to independently initiate tricks toward the end of training and following its conclusion, both inside and outside of research sessions, in the absence of instruction to play tricks. Still, future research may want to include multiple question topographies to ensure that responding is not under selective stimulus control and could train caregivers to collect data on whether participants independently emit trick-playing behaviors outside of sessions. Moreover, future research could consider fading out the presence of the experimenter. In addition, future research could assess the extent to which the current training procedure results in generalization of trick-playing skills to natural social interactions with peers outside of ABA sessions. Such data would also further evaluate the social validity of the intervention, from the standpoint of evaluating whether the procedure produced a socially meaningful behavior change in a fully generalized natural environment setting with peers.

Seventh, there was a varying number of trick trials presented across conditions and sessions. In particular, because trick playing was targeted during behavioral intervention sessions that included other skill acquisition programs, we decided to only conduct three trick trials during baseline sessions in an effort to avoid taking up more of the participants’ session time. On the other hand, during training, three to seven trick trials were presented each session, and the number of trick trials varied based on how many tricks were possible to conduct naturally during a behavioral intervention session given that the intricacy of each trick varied (some were short and others took more time to set up and carry out). Again, since trick-playing sessions occurred in the context of behavioral intervention sessions, there needed to be an adequate amount of time spent teaching other skill acquisition programs. Hence, the decision to conduct one to two other skill acquisition programs between each trick. From a methodological standpoint, the number of trick trials presented each session could be held constant in future research.

The choice of aggregating data across multiple tricks in a single session, rather than graphing each trick, is worthy of a brief discussion. Each trick trial produced data on correct or incorrect implementation of each of four component behaviors of playing a trick, therefore the percentage of trick playing behaviors for each trick trial could be scored as 0%, 25%, 75%, or 100% correct. One option would have been to graph these data for each trick trial as one data point, giving a more fine-grained analysis of trick-playing behavior for each trial. However, it should be noted that calculating percentage correct with a denominator of trials that is very low can be problematic (Cooper et al., 2019). Therefore, the choice was made to aggregate all trick trials per session into a calculation of percentage correct, yielding a denominator of trick-playing component behaviors of 12–28 for each session.

Eighth, it is unclear which components of the treatment package were responsible for behavior change, which could be eliminated, and whether or not it was necessary to initiate phase 1 of training (i.e., it may have been sufficient to require only phase 2 of training). Future research should conduct a component analysis to answer such questions.

Last, the measures of social validity would have been stronger if we included a measure of social validity from the participant’s perspective. For example, future research could include social validity questions for the participants to answer and/or directly measuring positive affect, such as laughing and smiling. Although no formal data were collected on affect, anecdotal observations consistently indicated that the participants smiled, laughed, and had fun during sessions. In addition, it should be noted that the training procedure in phase 2 had participant social validity built-in, in the sense that the participants created and chose which tricks they wanted to play; the researchers did not dictate which tricks to play.

This study provides further evidence that applied behavior analytic teaching procedures can be used to successfully teach complex social skills to Autistic individuals and that such training can be fun. In particular, deception was taught through the use of friendly tricks. In addition, the current study demonstrates that substantial changes in the overt behaviors involved in perspective taking can be achieved through applied behavior analytic teaching procedures.

## Data Availability

Data will be made available upon reasonable request.

## References

[CR1] Autistic Science Person. (2021). Be honest: Autistic vs. neurotypical honesty. https://autisticscienceperson.com/2021/05/17/be-honest-autistic-vs-neurotypical-honesty/. Accessed 9/1/2023

[CR2] Baron-Cohen, S. (1995). Mindblindness: An essay on autism and theory of mind. *Bradford Books/MIT Press*. 10.7551/mitpress/4635.001.0001

[CR3] Baron-Cohen, S., Leslie, A., & Frith, U. (1985). Does the autistic child have a theory of mind? *Cognition,**21*, 37–46. 10.1016/0010-0277(85)90022-8 2934210 10.1016/0010-0277(85)90022-8

[CR4] Bergstrom, R., Nadjowski, A., Alvarado, M., & Tarbox, J. (2016). Teaching children with autism to tell socially appropriate lies. *Journal of Applied Behavior Analysis,**49*(2), 405–410. 10.1002/jaba.295 26831011 10.1002/jaba.295

[CR5] Buijsman, R., Begeer, S., & Scheeren, A. M. (2022). “Autistic person” or ”person with autism”? Person-first language preference in Dutch adults with autism and parents. *Autism,**27*(3), 788–795. 10.1177/13623613221117914 35957517 10.1177/13623613221117914 PMC10074744

[CR6] Charlop-Christy, M. H., & Daneshvar, S. (2003). Using video modeling to teach perspective taking to children with autism. *Journal of Positive Behavior Interventions,**5*(1), 12–21. 10.1177/10983007030050010101

[CR7] Cooper, J. O., Heron, T. E., & Heward, W. L. (2019). *Applied behavior analysis* (3rd ed.). Hoboken, NJ: Pearson.

[CR8] Dhadwal, A. K., Najdowski, A. C., & Tarbox, J. (2021). A Systematic replication of teaching children with autism and other developmental disabilities correct responding to false-belief tasks. *Behavior Analysis in Practice,**14*(2), 378–386. 10.1007/s40617-020-00531-x 34150454 10.1007/s40617-020-00531-x PMC8149531

[CR9] Dixon, M. R., Belisle, J., Hayes, S. C., Stanley, C. R., Blevins, A., Gutknecht, K. F., Partlo, A., Ryan, L., & Lucas, C. (2021). Evidence from children with autism that derived relational responding is a generalized operant. *Behavior Analysis in Practice,**14*(2), 295–323. 10.1007/s40617-020-00425-y 34150448 10.1007/s40617-020-00425-y PMC8149511

[CR10] Erhard, P., El Fattal, R., & Van Etten, N. (2021). Multiple exemplar training. In F. R. Volkmar (Ed.), *Encyclopedia of autism spectrum disorders* (pp. 3043–3044). Springer. 10.1007/978-3-319-91280-6_102319

[CR11] Hayes, S. C., Barnes-Holmes, D., & Roche, B. (2001). Relational frame theory: A post-Skinnerian account of human language and cognition. *Kluwer Academic/Plenum*. 10.1016/S0065-2407(02)80063-5 10.1016/s0065-2407(02)80063-5 11605362

[CR12] Hogrefe, G. J., Wimmer, H., & Perner, J. (1986). Ignorance versus false belief: A developmental lag in attribution of epistemic states. *Child Development,**57*, 567–582. 10.2307/1130337

[CR13] LeBlanc, L. A., Coates, A. M., Daneshvar, S., Charlop-Christy, M. H., Morris, C., & Lancaster, B. M. (2003). Using video modeling and reinforcement to teach perspective-taking skills to children with autism. *Journal of Applied Behavior Analysis,**36*(2), 253–257. 10.1901/jaba.2003.36-253 12858990 10.1901/jaba.2003.36-253 PMC1284438

[CR14] Najdowski, A. C., Bergstrom, R., Tarbox, J., & St. Clair, M. (2017). Teaching children with autism to respond to disguised mands. *Journal of Applied Behavior Analysis,**50*(4), 733–743. 10.1002/jaba.413 28901545 10.1002/jaba.413

[CR15] Najdowski, A. C., & St. Clair, M., Fullen, J. A., Child, A., Persicke, A., & Tarbox, J. (2018). Teaching children with autism to identify and respond appropriately to the preferences of others during play. *Journal of Applied Behavior Analysis,**51*(4), 890–898. 10.1002/jaba.494 30006926 10.1002/jaba.494

[CR16] Perner, J., Frith, U., Leslie, A. M., & Leekam, S. R. (1989). Exploration of the autistic child’s theory of mind: Knowledge, belief, and communication. *Child Development,**60*, 689–700. 10.2307/1130734 2737018

[CR17] Perner, J., Leekam, S. R., & Wimmer, H. (1987). Three-year olds’ difficulty with false belief. The case for a conceptual deficit. *British Journal of Developmental Psychology,**5*, 125–137. 10.2307/1130760

[CR18] Persicke, A., Tarbox, J., Ranick, J., & Clair, M. S. (2013). Teaching children with autism to detect and respond to sarcasm. *Research in Autism Spectrum Disorders,**7*(1), 193–198. 10.1016/j.rasd.2012.08.005

[CR19] Reinecke, D. R., Newman, B., Kurtz, A. L., Ryan, C. S., & Hemmes, N. S. (1997). Teaching deception skills in a game-play context to three adolescents with autism. *Journal of Autism & Developmental Disabilities,**27*, 127–137. 10.1023/A:1025835706522 10.1023/a:1025835706522 9105964

[CR20] Russell, J., Mauthner, M., Sharp, S., & Tidswell, T. (1991). The “windows task” as a measure of strategic deception in preschoolers and autistic subjects. *British Journal of Developmental Psychology,**9*, 331–349. 10.1111/j.2044-835X.1991.tb00881.x

[CR21] Sage. (n.d.). Terminology guidance autism: *The International Journal of Research and Practice*. https://journals.sagepub.com/pb-assets/cmscontent/AUT/Autism-terminology-guidance-2021-1626860796.pdf. Accessed 9/1/2023

[CR22] Sauter, J. A., Stocco, C. S., Luczynski, K. C., & Moline, A. D. (2020). Temporary, inconsistent, and null effects of a moral story and instruction on honesty. *Journal of Applied Behavior Analysis,**53*(1), 134–146. 10.1002/jaba.552 30874313 10.1002/jaba.552

[CR23] Skinner, B. F. (1969). *Contingencies of reinforcement: A theoretical analysis.* Appleton-Century-Crofts.

[CR24] Sodian, B., & Frith, U. (1992). Deception and sabotage in autistic, retarded and normal children. *Journal of Child Psychology & Psychiatry,**33*, 591–605. 10.1111/j.1469-7610.1992.tb00893.x 1577901 10.1111/j.1469-7610.1992.tb00893.x

[CR25] St. Clair, M., Najdowski, A. C., Welsh, F., Simchoni, L., Milne, C. M., Fullen, J. A., Acuña, B., & Suarez, V. D. (2022). Teaching children with autism to identify known and unknown information across self and others. *Behavior Analysis in Practice,**16*(3), 837–848. 10.1007/s40617-022-00768-8 37680330 10.1007/s40617-022-00768-8 PMC10480121

[CR26] Stocco, C. S., Moline, A. D., & Bowar, S. (2021). Further evaluation of contingencies on lying about homework completion. *Behavioral Interventions,**36*(3), 620–634. 10.1002/bin.1787

[CR27] Suarez, V. D., Najdowski, A. C., Tarbox, J., Moon, E. I., & St. Clair, M., & Farag, P. (2021). Teaching children with autism problem-solving skills for resolving social conflicts. *Behavior Analysis in Practice,**15*(3), 768–781. 10.1007/s40617-021-00643-y 34484617 10.1007/s40617-021-00643-y PMC8404753

[CR28] Talwar, V., Zwaigenbaum, L., Goulden, K. J., Manji, S., Loomes, C., & Rasmussen, C. (2012). Lie-telling behavior in children with autism and its relation to false-belief understanding. *Focus on Autism & Other Developmental Disabilities,**27*(2), 122–129. 10.1177/1088357612441828

[CR29] Tarbox, J., Campbell, V., & Pio, S. (2020). Rule governed behavior and verbal regulation. In R. A. Rehfeldt, J. Tarbox, M. Fryling, & L. Hayes (Eds.), *Applied behavior analysis of language and cognition* (pp. 214–233). New Harbinger.

[CR30] Welsh, F., Najdowski, A. C., Strauss, D., Gallegos, L., & Fullen, J. A. (2019). Teaching a perspective-taking component skill to children with autism in the natural environment. *Journal of Applied Behavior Analysis*, *52*(2), 439–450. 10.1002/jaba.523 10.1002/jaba.523 30461010

