# Correction: Making Deception Fun: Teaching Autistic Individuals How to Play Friendly Tricks

**DOI:** 10.1007/s40617-024-00955-9

**Published:** 2024-05-02

**Authors:** Megan St. Clair, Kacie Massoudi, Jonathan Tarbox, Adel Najdowski, Lauri Simchoni, Marianne Jackson, Angela Persicke

**Affiliations:** 1 Chicago School of Professional Psychology, Los Angeles, CA USA; 2 California State University, Fresno, CA USA; 3 Center for Autism and Related Disorders, Inc, Woodland Hills, Los Angeles, CA USA; 4 Halo Behavioral Health, Sherman Oaks, CA USA; 5 University of Southern California, Los Angeles, CA USA; 6 Pepperdine University, Malibu, CA USA

**Correction: Behavior Analysis in Practice**

10.1007/s40617-024-00935-z

This article was updated to correct Adel Najdowski's name from Adel Nadjowski.

